# A Proteomic Study of Human Merkel Cell Carcinoma

**DOI:** 10.4172/jpb.1000291

**Published:** 2013-11-25

**Authors:** Qiang Shao, Stephanie D. Byrum, Linley E. Moreland, Samuel G. Mackintosh, Aarthi Kannan, Zhenyu Lin, Michael Morgan, Brendan C. Stack, Lynn A. Cornelius, Alan J. Tackett, Ling Gao

**Affiliations:** 1 Department of Dermatology, University of Arkansas for Medical Sciences, 4301 W. Markham St., Little Rock, AR 72205, USA; 2 Department of Biochemistry and Molecular Biology, University of Arkansas for Medical Sciences, 4301 W. Markham St., Little Rock, AR 72205, USA; 3 University of South Florida, College of Medicine, Tampa, FL, USA; 4 Department of Otolaryngology-Head and Neck Surgery, University of Arkansas for Medical Sciences, 4301 W. Markham St., Little Rock, AR 72205, USA; 5 Department of Internal Medicine, Division of Dermatology, Washington University School of Medicine in St. Louis, 63110, USA; 6 Critical Care Medicine, the First Affiliated Hospital of Nanchang University, Nanchang, Jiangxi, China; 7 Cancer Center Union Hospital, Tongji Medical College, Huazhong University of Science and Technology, Wuhan, China

**Keywords:** Merkel cell carcinoma, PI3K/mTOR pathway, Liquid tissue platform

## Abstract

Merkel Cell Carcinoma (MCC) is an aggressive neuroendocrine cancer of the skin. The incidence has been quadrupled with a 5-year mortality rate of 46%, presently there is no cure for metastatic disease. Despite the contribution of Merkel cell polyomavirus, the molecular events of MCC carcinogenesis are poorly defined. To better understand MCC carcinogensis, we have performed the first quantitative proteomic comparison of formalin-fixed, paraffin-embedded (FFPE) MCC tissues using another neuroendocrine tumor (carcinoid tumor of the lung) as controls. Bioinformatic analysis of the proteomic data has revealed that MCCs carry distinct protein expression patterns. Further analysis of significantly over-expressed proteins suggested the involvement of MAPK, PI3K/Akt/mTOR, wnt, and apoptosis signaling pathways. Our previous study and that from others have shown mTOR activation in MCCs. Therefore, we have focused on two downstream molecules of the mTOR pathway, lactate dehydrogenase B (LDHB) and heterogeneous ribonucleoprotein F (hnRNPF). We confirm over-expression of LDHB and hnRNPF in two primary human MCC cell lines, 16 fresh tumors, and in the majority of 80 tissue microarray samples. Moreover, mTOR inhibition suppresses LDHB and hnRNPF expression in MCC cells. The results of the current study provide insight into MCC carcinogenesis and provide rationale for mTOR inhibition in pre-clinical studies.

## Introduction

Merkel cell carcinoma (MCC) is an aggressive neuroendocrine cancer of the skin with a quadrupled incidence in the past 15 years. The mortality rate is 46%, exceeding that of melanoma, and there is presently no cure. Moreover, its incidence is approximately 11-fold in AIDS patients and 5-fold in organ transplant patients. In addition to skin cancers, patients with MCC have increased risk for multiple myeloma, non-Hodgkin’s lymphoma, and in particular chronic lymphocytic leukemia. Although chronic sun exposure, polyomavirus and immunosuppression have been implicated in the tumor development [1–4], our understanding of the cellular and molecular mechanisms of MCC carcinogenesis and metastasis remains largely unknown.

Interrogation of MCC tumors of mutation of both tumor suppressor genes and oncogenes, such as p53, PTEN, Ras, B-RAF, c-kit, β-catenin, which are frequently involved in human cancers, have failed to reveal a significant role in MCC [5]. However, loss of the pRb1 gene region and amplification of the L-Myc gene region have been found at a significant rate (26% and 31% of tumors, respectively) and have been postulated to have a functional role in tumor development [6]. In search of receptor tyrosine kinase (RTK) involvement in MCC (and a rationale for the use of targeted therapies), studies have found variable expression of c-kit, VEGFs, PDGFα and PDGFβ in MCCs compared to normal skin [7,8]. Moreover, study has shown MAP kinase pathway is silent (as demonstrated by lack of pathway activation and no ERK phosphorylation) in the majority of MCCs examined [9]. Furthermore, a separate study using a MCC cell line demonstrates that inactivation of MAP kinase pathway is important in MCC carcinogenesis [10]. Additionally, one study using tissue microarray shows expressions of MMPs, VEGFs, P38, stromal NF-Kappa B and synaptophysin are associated with aggressive behavior [11].

Genomic studies such as chromosomal comparative genomic hybridization (CGH) have been employed to examine copy number alterations in MCCs. Chromosomes 1, 3q, 5p and 6 are frequently increased in copy number whereas chromosomes 3p, 4, 5q, 7, 10 and 13 are frequently lost [12]. Additionally, transcriptome profiling has identified a subgroup of MCCs with intratumoral CD8 positive T cell infiltration that is associated with better prognosis [13]. Although the causes of cancer lie in mutations or epigenetic changes at the chromosomal level, their molecular manifestation is correlated to the dysfunction of biochemical pathways at the protein level. In addition, the plasticity of mRNAs raises questions whether RNA expression changes are translated to those of proteins that are central to carcinogenesis. Therefore, defining the protein profiles and dysregulation of their expression level in cancer is critical.

Global proteomic analysis has become a promising strategy to identify potential biomarkers in various cancer subtypes. However, one of the obstacles of human tissue research for proteomic study is the preferential use of snap frozen fresh tissues that are restricted in human skin biopsy samples. The Liquid Tissue platform, a novel technology for protein extraction from formalin-fixed, paraffin-embedded (FFPE) tissue blocks, permits facile global proteomic analysis of archival specimens by mass spectrometry to identify novel or critical proteins from human archival tissues. Moreover, no proteomic study has been performed in MCC and the proteins essential for the transformation of MCC have not been identified.

In this study, we used a quantitative proteomic platform to assessprotein expression in FFPE MCC tumors. Because of the neuroendocrine nature of MCC, we chose another neuroendocrine tumor, carcinoid tumors of the lung, as the control. We identified significantly over-expressed proteins in MCC. Interestingly, further pathway analysis of our protein data implicated the involvement of MAPK, PI3K/Akt/mTOR, wnt, and apoptosis signaling pathways. As shown previously mTOR pathway is activated in MCCs [14,15], therefore we selected this pathway for further investigation. Two molecules downstream of the mTOR pathway, lactate dehydrogenase B (LDHB) and heterogeneous ribonucleoprotein F (hnRNPF), were studied. We first confirmed the expression of LDHB and hnRNPF in tissue microarray including 80 MCC samples and two primary human MCC cell lines established in the lab. Moreover, mTOR inhibition suppressed both LDHB and hnRNPF expression in MCC cells. The results of the current study will provide insight into our understanding of MCC carcinogenesis and has translational potential for clinical practice by facilitating the identification of useful biomarkers for early diagnosis and prognosis as well as identifying novel therapeutic targets of MCC.

## Materials and Methods

### Sample selection and tissue microarray

In accordance with institutional approvals for human study protocol, a total of 10 MCCs and 5 carcinoid tumor of the lung formalin fixed paraffin embedded (FFPE) tissue blocks were selected for proteomic study. Tissue microarray (TMA) included 80 FFPE MCC tissue blocks and were prepared as previously described [15]. Briefly, for each case a representative area from the tumor was carefully selected from a hematoxylin-eosin stained section of a MCC tissue block. Core cylinders (0.6 mm) were punched from each FFPE tumor and deposited into a recipient paraffin block using the MTA-I manual tissue arrayer (Beecher). Five-micrometer sections of the resulting TMA blocks were made and used for immunohistochemistry.

### Immunohistochemistry

Immunohistochemistry was performed on 5 μm sections of TMA slides. The slides were deparaffinized and rehydrated in water. Antigen retrieval was performed by microwaving in 0.01 M sodium citrate for 20 min. Tissue peroxide activity was blocked with 1% hydrogen peroxide at room temperature (RT) for one hour followed by washing twice in PBS. The sections were further blocked with normal goat serum at RT for one hour followed by incubation with LDHB (Lifespan Biosciences) and hnRNPF (Abcam) at 4°C overnight, respectively. Secondary goat anti-rabbit antibody (1:200) was applied to the slides for one hour at RT before developing in HRP detection system and freshly prepared diaminobenzidine as the chromogen (brown). Sections were counterstained with hematoxylin. Staining was manually scored. Immunostained slides were viewed on an Olympus BX51 Research System Microscope by 10× and 20× UPlanApo air objective lenses (Olympus America). Images were photographed using a high-resolution interline CCD camera (CoolSNAP*cf*, Photometrics), and acquired with automated microscopy acquisition software (MetaMorph version 7.7, Molecular Devices).

### Cell lines and reagents

In accordance with institutional approvals for human study protocol, we have established two primary human Merkel cell carcinoma cell lines (MCC-2 and MCC-3) from lymph node metastases of two patients [15]. Both cell lines were maintained in RPMI medium with 10% Fetal Bovine Serum (FBS), penicillin and streptomycin. Fresh medium was added every other day and cultures were split 1:2 weekly following complete removal of the medium. mTOR inhibitors Ku- 0063784 and PP242 were obtained from Sigma Aldrich.

### Immunoblotting

Membranes were blotted with antibodies directed against (LDHB and hnRNPF). Bound antibodies were detected with horseradish peroxidase-linked antibody against mouse or antibody against rabbit (IgG; Amersham), followed by ECL detection (Amersherm).

### Gene expression analysis

RNAs were isolated from MCC fresh tissues and control carcinoid tumors of the lung with RNeasy kit (Qiagen). cDNA was generated from mRNA using a Reverse Transcription Kit (Applied Biosystems). SYBR Green-based quantitative reverse transcription-PCR (qRT-PCR) was performed with a StepOnePlus Real-Time PCR System (Applied Biosystems). Triplicate runs of each sample were normalized to MRPS2 mRNA to determine relative expression.

### Quantitative proteomics

A single 10 μm tissue section was made and mounted on Director slide (Expression Pathology, Gaithersburg, MD), and heated for 1 hour at 60°C. Paraffin was removed with xylene followed by tissue rehydration through a series of graded ethanol solutions and distilled water. Approximately 30,000 tumor cells were procured by needle microdissection.

The Liquid Tissue MS Protein Prep Kit (Expression Pathology) was used to reverse cross-linking and the extracted proteins were analyzed by Coomassie/sodium dodecyl sulfate polyacrylamide gel electrophoresis (SDS-PAGE). Each SDS-PAGE gel lane was cut into 3 mm slices and subjected to in-gel trypsin digestion as follows. Protein-containing gel slices were destained in 50% methanol (Fisher), 100 mM ammonium bicarbonate (Sigma-Aldrich), followed by reduction in 10 mM Tris [2-carboxyethyl]phosphine (Pierce) and alkylation in 50 mM iodoacetamide (Sigma-Aldrich). Gel slices were then dehydrated in acetonitrile (Fisher), followed by addition of 100 ng porcine trypsin (Promega) in 100 mM ammonium bicarbonate (Sigma-Aldrich) and incubation at 37°C for 12–16 hours. Peptide products were then acidified in 0.1% formic acid (Fluka). Tryptic peptides were separated by reverse phase Jupiter Proteo resin (Phenomenex) on a 100×0.1 mm column using a nanoLC 2D system (Eksigent). Peptides were eluted using a 30 min gradient from 98:2 to 40:60 buffer A:B ratio. [Buffer A=0.1% formic acid, 0.05% acetonitrile; buffer B=0.1% formic acid, 75% acetonitrile.] Eluted peptides were ionized by electrospray (2.0 kV) followed by MS/MS analysis using collision induced dissociation on an LTQ XL mass spectrometer (Thermo). MS data were acquired over a range of 375 to 1500 m/z. MS/MS data were acquired for the top 7 peaks from each MS scan. Proteins were identified by database search using Mascot (Matrix Science). Tandem mass spectrometric data was searched with an in-house version of Mascot against the UniprotKB/SwissProt *Homo sapiens* protein database for protein identification. In order to quantify the relative protein level in these samples, we used a mass spectrometric technique called spectral counting using parameters as detailed by Byrum et al. [16]. A spectral count is the number of tandem mass spectra assigned to a given protein and reflects the abundance of the protein. We then calculated a normalized spectral abundance factor (NASF), which reflects the amount of a given protein relative to the total proteins identified in the gel lane [17,18]. The NASF was calculated as follows:

$${\left(\mathrm{NASF}\right)}_{k}=\frac{(\frac{\mathrm{SpC}}{L})k}{\sum_{i=1}^{N}(\frac{\mathrm{SpC}}{L})i}$$

The variables are defined as follows: k is a given protein, SpC are the spectral counts, L is the length of the protein, and N is the sum of all proteins identified in the gel lane. For a given protein, this reveals what fraction of the total proteins identified in the gel lane is the particular protein. The data distribution of the normalized spectral counts showed a bimodal distribution and therefore, the Wilcoxon rank sum test with the t-approximation was used to identify significantly differentiating proteins between the two groups. The enrichment level for each protein was identified by calculating the fold change (CK/Lung) using the average ln (NSAF) values for each protein. Fold change was calculated by taking the anti-log of (ln(NSAF)_avg CK_−ln(NSAF)_avg Lung_). Proteins with a *p*-value<0.05 and a FC>1.5 were considered significant.

The most important signaling pathways were identified using the Database for Annotation, Visualization and Integrated Discovery (DAVID) v6.7 [19]. Significantly differentiating proteins, not identified in signaling pathways by DAVID, were searched in the literature using a web-based search tool, PubTator, for involvement in known pathways using the protein’s gene symbol plus the keyword “pathway” [20,21].

## Results

### Distinct protein expression profiles in Merkel cell carcinoma

**Figure 1:**
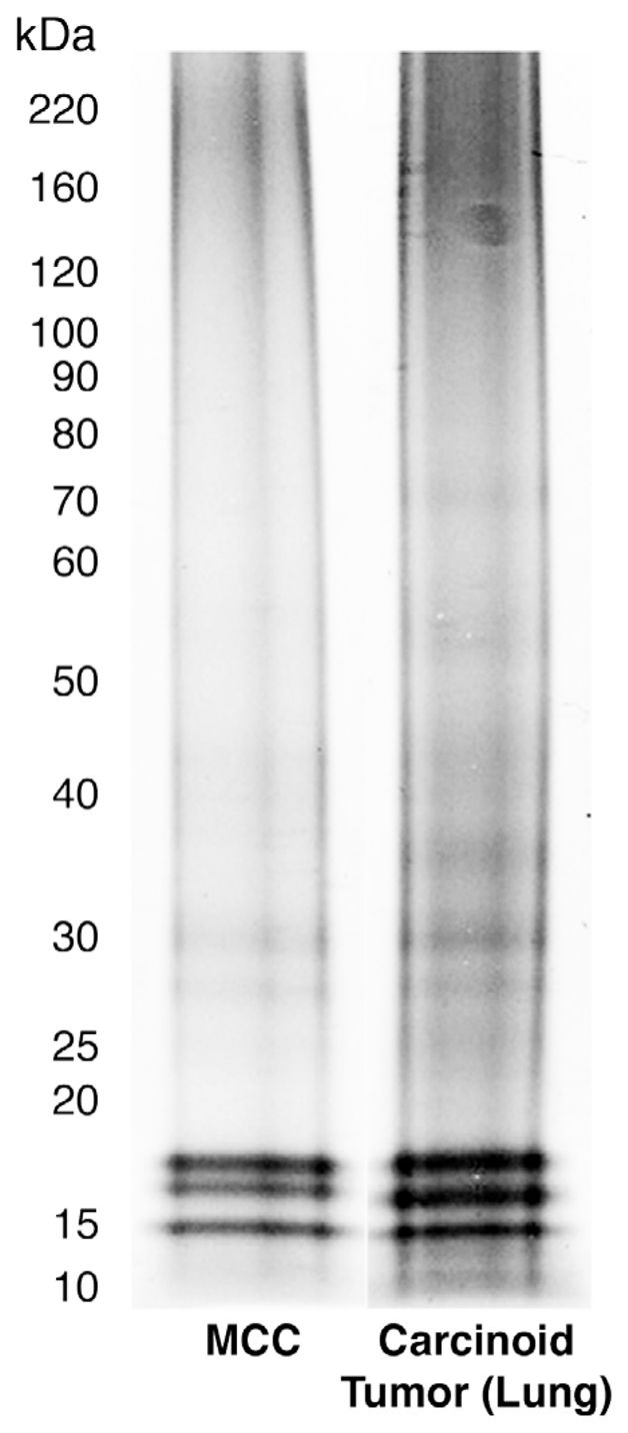
Representative gel lanes for quantitative mass spectrometric analysis of MCC and carcinoid tumor of the lung from formalin-fixed paraffin-embedded (FFPE) tissues Proteins were extracted from 10 MCC FFPE tissues and 5 carcinoid tumors of the lung FFPE tissues. The samples were equally loaded and resolved by sodium dodecyl sulfate polyacrylamid gel electrophoresis (SDS-PAGE)/Coomassie. Each gel lane was sliced into 23 bands, subjected to trypsin digestion and peptides were analyzed by liquid chromatography-mass spectrometry (LC-MS)/MS with a Thermo LTQ-XL mass spectrometer. Proteins were identified by a Mascot database search (95% confidence threshold). One gel lane from each group is shown.

**Figure 2:**
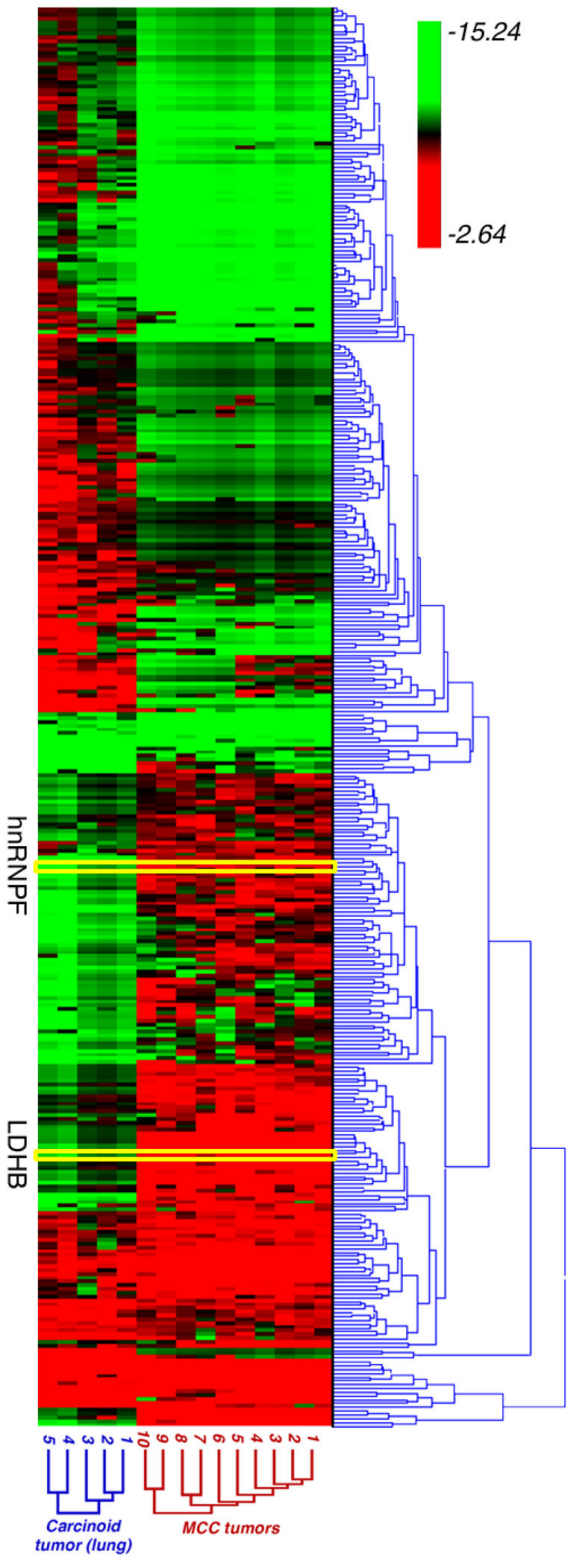
Hierarchical clustered heat map of the 375 significant proteins differentially expressed between MCC and carcinoid tumors of the lung as determined by Wilcoxon rank sum test (p < 0.05) LDHB and hnRNPF proteins were upregulated in MCC tumors compared to carcinoid tumors of the lung and were selected for further validation.

The proteome from 10 metastatic MCC tumors and 5 carcinoid tumor of the lung were measured in this study. As shown in Figure 1, each protein sample was resolved by Coomassie/SDS-PAGE followed by in-gel trypsin digestion and LC-MS/MS. A total of 1356 proteins were identified for all samples at a 1% false discovery rate using a decoy database. To determine whether a protein was differentially expressed between MCC and the carcinoid tumors of the lung, a label-free approach based on spectral counting was used [18,22–24]. The relative abundance of each protein was normalized using the normalized spectral abundance factor (NSAF) and the frequency distribution of ln(NSAF) values showed a bimodal distribution. There were a total of 432 proteins identified with a fold change>1.5 in MCCs compared to the carcinoid tumor of lung. A Wilcoxon rank sum test with t-approximation identified 375 significantly differentiating proteins between MCCs and carcinoid tumor of the lung with a p-value<0.05. A heat map was generated using Hierarchical Clustering Explorer (HCE version 3.0) with all 375 significant proteins, the average linkage method, and Euclidean distance metric. The MCC and carcinoid tumor of the lung patient samples were clearly separated into two separate clusters based on these significantly differentiating proteins. Up- or down-regulated proteins in MCCs are indicated in red and green, respectively (Figure 2).

Pathway analysis using DAVID uncovered several signaling pathways that are potentially important for MCC pathogenesis (Table 1). Proteins identified are also known to play a role in multiple diseases such as Parkinson’s disease, Huntington’s disease, systemic lupus erythematosus, and are associated with normal metabolic activities such as the citrate cycle, glycolysis/gluconeogenesis, and metabolism of amino acids. Of particular interest are proteins involved in focal adhesion, epithelial cell signaling, and the spliceosome. A manual literature search using the significantly differentiatingproteins in PubTatorhas revealed proteins involved in MAPK, PI3K/Akt/mTOR, wnt, and apoptosis signaling pathways (Table 2). Interestingly, we have uncovered several proteins involved in the MAPK pathway, which has previously been indicated as silent in MCCs [10]. The PI3K/AKT/mTOR signaling cascade is commonly dysregulated in human cancers [25–27]. Moreover, we have found mTOR activation is common in MCCs [15]. Therefore, we have selected two proteins, LDHB and hnRNPF, which are found to be significantly up-regulated in MCCs and are downstream effectors of mTOR pathways, for further study and validation.

### Expressions of LDHB and hnRNP are up-regulated in fresh tumor tissues and primary human MCC cell lines at the mRNA level

The mammalian target of rapamycin (mTOR) pathway is a master regulator of protein synthesis and frequently activated in human cancers [28]. mTOR resides in two complexes, mTOR complex1 (mTORC1) and mTOR complex (mTORC2), which execute distinct cellular tasks. Rapamycin and its analogues are allosteric inhibitors via mTORC1 inhibition. Underscored by the clinical inefficacy of allosteric inhibitors, more potent inhibitors of the active site of mTOR kinase, such as PP242 and Ku-0063794, have been developed. The major regulators of protein synthesis downstream of mTOR are eukaryotic translation initiation factor 4E (eIF4E)-binding protein 1 (4E-BP1) and S6 kinase (S6K). Similar to 4E-BP1, dysregulation of S6K signaling has been linked to human pathologies, including cancer and diabetes. There are two isoforms, S6K1 and S6K2, which are found to be up-regulated at both the RNA and protein levels in several types of human cancers. In contrast to S6K1, the S6K2 is specially associated with a number of RNA-binding proteins, including heterogeneous ribonucleoproteins (hnRNPs). Moreover, hnRNPF has been shown to regulate cell proliferation via S6K2 in breast cancer cell lines [29].

In tumor cells, glucose is preferentially converted into lactic acid through aerobic glycolysis, which is known as the “Warburg effect”. LDH is the key glycolytic enzyme catalyzing the formation of lactic acid from pyruvate, is often activated in cancers [30]. LDHB is critical for hyperactive mTOR mediated tumorigenesis.

**Figure 3:**
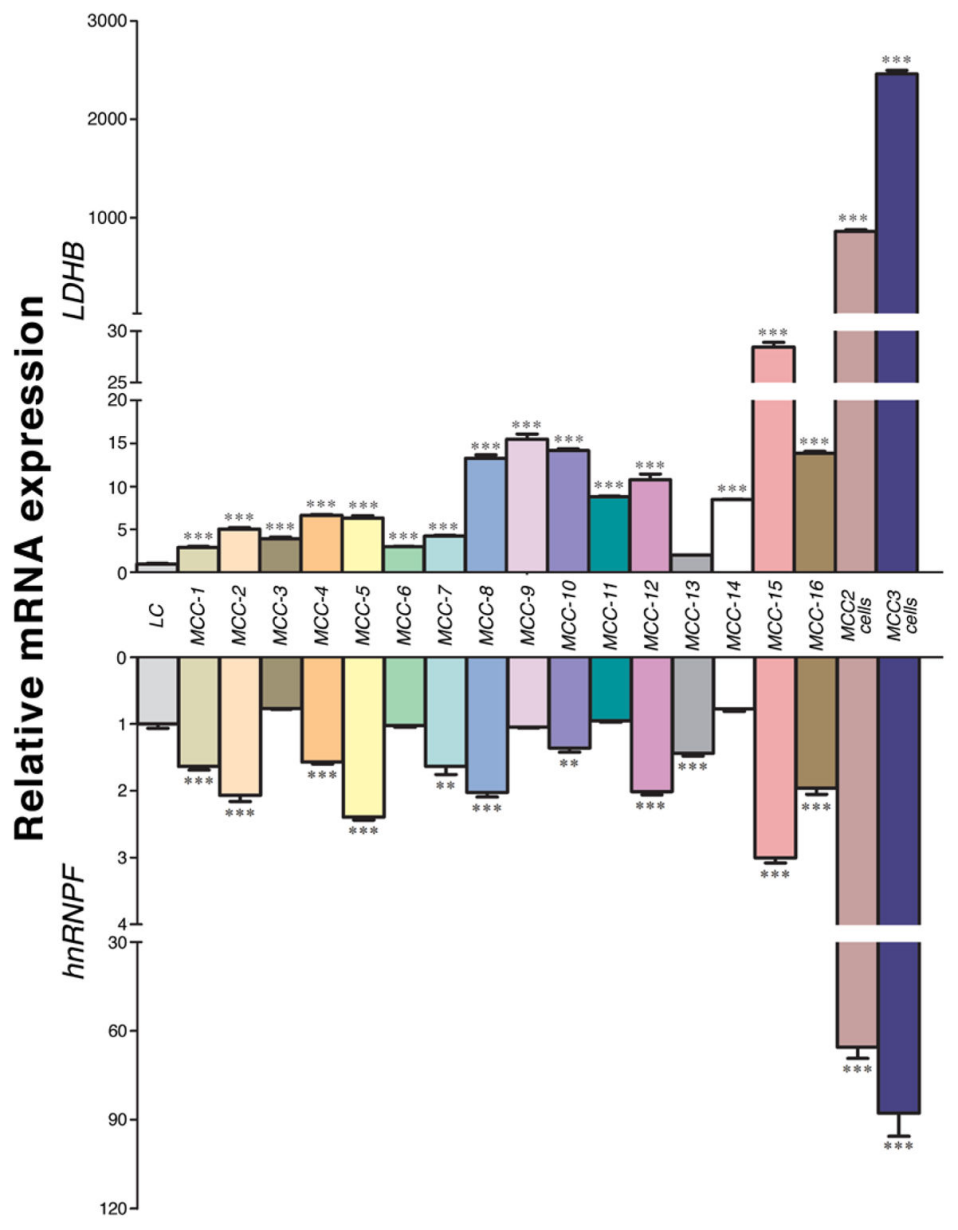
Expression of LDHB and hnRNPF in fresh MCC tumor tissues and two primary human MCC cell lines cDNAs were extracted from 16 fresh MCC tumors and 2 primary human MCC cell lines and qRT-PCR analysis of LDHB and hnRNPF mRNA expression was performed. cDNA from a fresh carcinoid tumor of the lung was used as a control. Triplicate runs of each sample were normalized to MRPS2 mRNA to determine relative expression (means ± SEM), (**P < 0.01, ***P< 0.001).

Taking advantage of two primary human MCC cells lines established in our laboratory as well as fresh MCC tumor samples, we measured the expression of LDHB and hnRNPF at the mRNA level. mRNAs were extracted from fresh tumors, MCC-2 and MCC-3 cell lines followed by qPCR. cDNA from a fresh carcinoid tumor of the lung was used as a control. Compared to the carcinoid tumor of the lung, significantly increased expression of LDHB and hnRNPF was found in 16/16 and 11/16 fresh MCC tumors, respectively (Figure 3). Similarly, both MCC-2 and MCC-3 cell lines demonstrated increased expression of both mRNAs. Therefore, we have demonstrated overexpression of LDHB and hnRNPF at the mRNA level in MCCs, which confirms our proteomic results.

### LDHB and hnRNPF are over-expressed in human MCC tissue microarray samples

**Figure 4:**
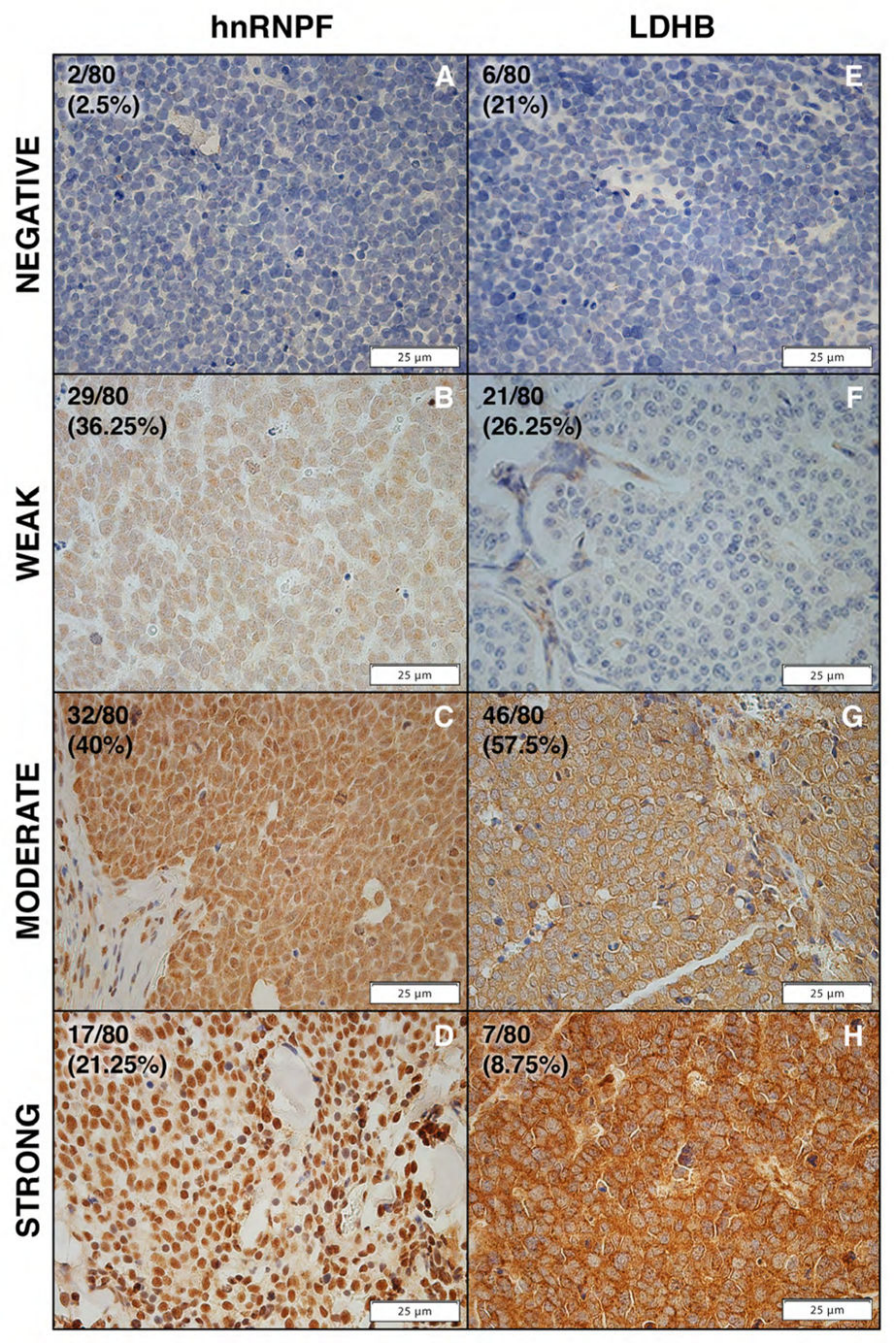
hnRNPF and LDHB expression in MCC tissue microarray samples (A–D) Representative negative and positive immunohistochemical staining of hnRNPF (brown nuclear staining) and percentages of negative samples, and samples with weak, moderate and strong positives. (E–H) Representative negative and positive immunohistochemical staining of LDHB (brown cytoplasmic staining) and percentages of negative samples, and samples with weak, moderate and strong positives.

To further confirm our observations, we examined 80 MCC tumor samples using TMA. As shown in Figure 4, over-expression as indicated by positive nuclear staining of hnRNPF (brown) and positive cytoplasmic staining of LDHB (brown), respectively. Over-expression of LDHB and hnRNPF was detected in the majority of MCCs. Seventy-four out of eighty (92.5%) and seventy-eight out of eighty (97.5%) MCC samples were positive for LDHB and hnRNPF, respectively. Thus, we have shown up-regulation of LDHB and hnRNPF in MCCs at the protein level, which again confirms our proteomic studies.

### mTOR inhibition suppresses LDHB and hnRNPF expressions in both MCC-2 and MCC-3 cells

**Figure 5:**
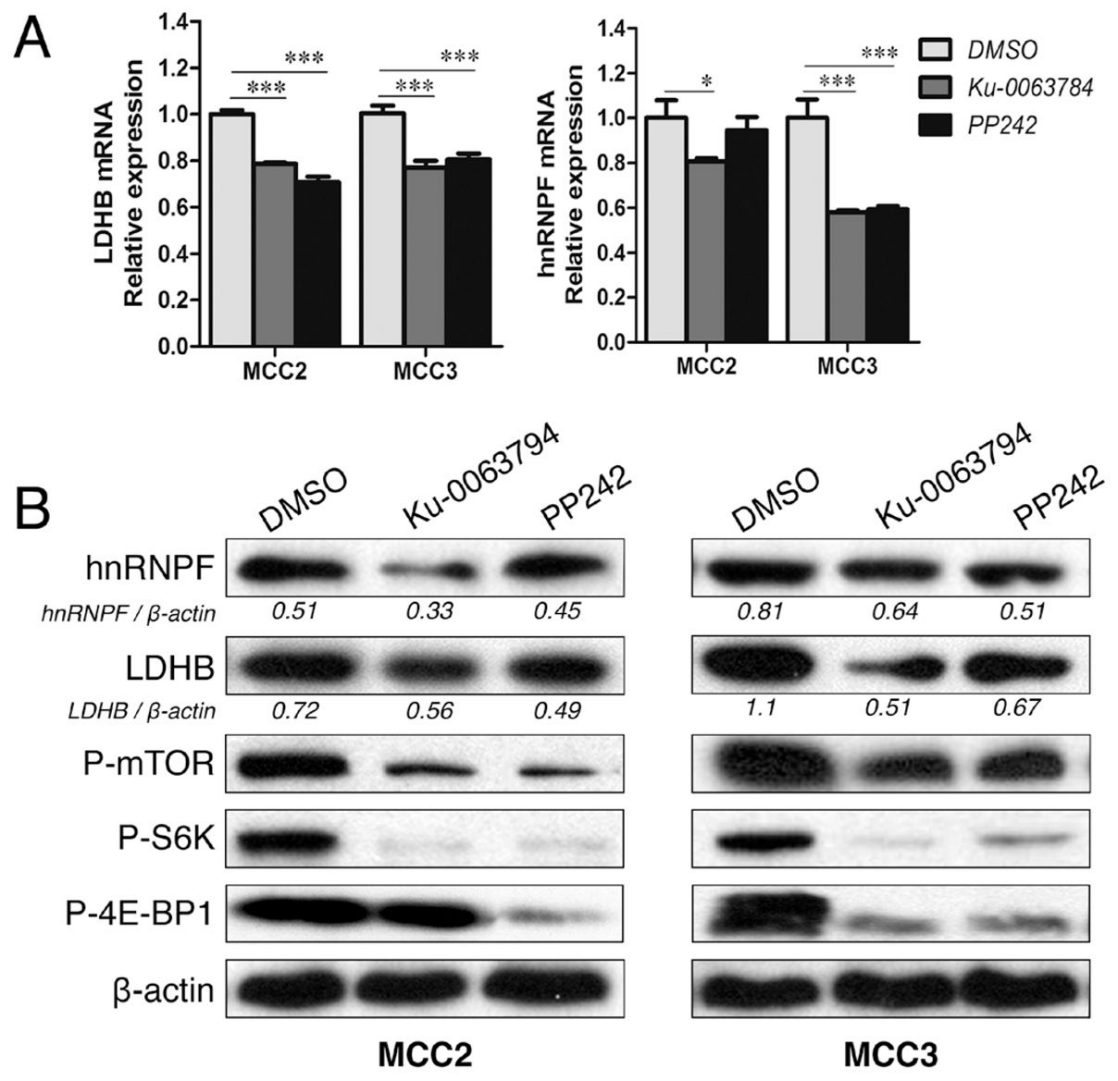
mTOR inhibition suppresses LDHB and hnRNPF expression in both MCC-2 and MCC-3 cells (A) Suppressed LDHB and hnRNPF mRNA expression in MCC-2 and MCC-3 cells. MCC-2 and MCC-3 cells were treated with DMSO, Ku-0063794 (5μM) and PP242 (2.5μM) for 24 hours, respectively. cDNAs were extracted and expression of hnRNPF and LDHB was analyzied by qPCR. Triplicate runs of each sample were normalized to MRPS2 mRNA to determine relative expression (means ± SEM), (^*^P < 0.05, ^***^P< 0.001). (B) Suppressed mTOR pathway, LDHB and hnRNPF protein expressions in MCC-2 and MCC-3 cells by immunoblottings. MCC-2 and MCC-3 cells were treated with DMSO, Ku-0063794 (5μM) and PP242 (2.5μM) for 24 hours, respectively. Lysates were prepared and subjected to immunoblotting analysis with indicated antibodies. β-actin served as proper loading control.

To evaluate whether LDHB and hnRNPF are downstream effectors of the mTOR pathway, MCC-2 and MCC-3 cellswere treated with the active site ATP mTOR inhibitors Ku-0063784 and PP242 for 24 hours, respectively. Cell lysates were subjected to LDHB and hnRNPF immunoblotting. mRNAs were extracted from MCC-2 and MCC-3 cells treated with mTOR inhibitors followed by qPCR analysis of LDHB and hnRNPF. Consistent with previous findings in other types of human cancer [29,30], reduced LDHB and hnRNPF expressions were observedboth at the mRNA and protein levels in MCC-2 and MCC-3 cells following mTOR pathway inhibition, indicating that LDHB and hnRNPF are downstream effectors of mTOR pathway (Figure 5).

## Discussion

A hallmark of human cancer is heterogeneity. At the genetic level, it reflects the complex series of changes resulting in the activation of oncogenes coupled with inactivation of tumor suppressor genes. At the patient level, it manifests by disease outcome, response to therapy and ability to metastasize. At the pathological level, it is observed where certain histological features are associated with more aggressive cancers. An ability to model this complexity is crucial to identify therapeutic targets for cancers evading therapy. However, targeted therapy at the gene level remains a challenge as there is a distinction between driver mutations that can propel the development of cancer and driver mutations on which the cancer cell continually depends. Interrogation of tumors at the genomic and transcriptomic level may therefore not precisely present the complexity of the tumor itself or its biologic environment, including fluctuating in clonal variation, changes in in gene expression and host response, and signaling events that lead to changes at the protein level. Because many drugs act on protein effectors, in combination with genomic and transcriptomic profiling, proteomic profiling offers the promise of additional insights into cancer status and may be a better approach to identify therapeutic targets.

Merkel cell carcinoma is a neuroendocrine skin tumor with aggressive behavior and poor prognosis. Fifty percent of patients are metastatic upon diagnosis. Despite standard treatment that is surgery followed by radiation therapy, one third of patients eventuate distant metastasis. Evidence-based effective chemotherapy for metastatic disease has not yet developed. We have taken the approach to investigate metastatic tissue, with the rationale that the tumor cells present in lymph node are molecularly programmed to “escape” surveillance mechanisms and metastasize – the ultimate behavior that drives disease progression. In this study, taking advantage of the liquid platform technique using archival tissues to identify molecules that are integral to specific signaling pathways known to be important in tumor biology. We have identified increased expression of members of the RTK/PI3K/Akt/mTOR, wnt and MAPK pathways, which we propose are important in MCC pathogenesis. In fact, we have confirmed the overexpression of LDHB and hnRNPF, two downstream mTOR effectors in MCCs by qPCR and immunoblotting, supporting the involvement of this pathway in MCC tumorogenesis.

Increased expression of LDHB in MCCs suggests increased tumor metabolism depending on glycolysis in energy demand and further implies the idea that MCCs are sensitive to perturbation in the end stage of glycolysis-lactate production, and thus open a therapeutic window in the clinics. Moreover, increased expression of hnRNPF is indicative of additional molecules along mTOR pathway other than 4E-BP1 and S6K1 involved in MCC pathogenesis. Aberrant RTK/PI3K/Akt/mTOR pathway has a key role in human cancer initiation, progression, invasion, metastasis and resistance to therapy. Our current study supports the findings of two separate independent studies, which has revealed a dysregulated RTK/PI3K/Akt/mTOR pathway in MCC [25,26]. Cumulatively, these findings suggest that inhibitors of this pathway hold treatment promise in these tumors, either as initial single agents, or in combination with standard cytotoxic chemotherapy and radiotherapy.

## Figures and Tables

**Table 1:** Signaling pathways identified by DAVID The list of significantly differentiating proteins between MCC and carcinoid tumors of the lung was imported into the DAVID functional annotation web-tool. The proteins identified in signaling pathways from the KEGG database are listed.

Pathway	Count	P-value	Proteins	Fold Enrichment	Bonferroni	Benjamini	FDR
**Oxidative phosphorylation**	24	8.09E-11	UQCRC1, ATP6AP1, ATP6V1G1, COX5A, UQCRFS1, COX5B, UQCRQ, UQCRFSL1, NDUFS7, NDUFS6, NDUFS4, ATP5L, ATP6V0D1, ATP5I, NDUFS1, NDUFA5, NDUFA2, ATP5F1, ATP6V1H, ATP6V1F, COX6C, NDUFV1, ATP6V1E1, ATP6V0A1, UQCRB	5.18657	1.00E-08	1.00E-08	9.34E-08
**Parkinson’s disease**	18	2.00E-06	NDUFA5, NDUFA2, UQCRC1, SLC25A4, ATP5F1, COX5A, VDAC2, UQCRFS1, UQCRQ, COX5B, UQCRFSL1, COX6C, VDAC1, NDUFS7, NDUFS6, NDUFS4, NDUFV1, NDUFS1, UQCRB	3.950708	2.48E-04	1.24E-04	0.00231
**Huntington’s disease**	19	5.51E-05	NDUFA5, NDUFA2, UQCRC1, SLC25A4, ATP5F1, COX5A, VDAC2, UQCRFS1, UQCRQ, COX5B, UQCRFSL1, COX6C, VDAC1, DCTN2, NDUFS7, NDUFS6, NDUFS4, NDUFV1, NDUFS1, UQCRB	2.96547	0.006812	0.002276	0.063633
**Systemic lupus erythematosus**	13	1.68E-04	HLA-DRB1, ACTN4, C4A, SNRPD3, ACTN1, HIST1H2BO, TROVE2, HIST1H2BL, HIST1H3A, H2AFY2, SNRPB, H2AFY, H3F3A	3.689101	0.020611	0.005193	0.193756
**Vibrio cholerae infection**	9	6.99E-04	ACTG1, ACTB, ATP6AP1, ATP6V1E1, ATP6V1H, ATP6V0A1, ATP6V1G1, ATP6V0D1, ATP6V1F	4.515095	0.083065	0.017194	0.804307
**Spliceosome**	13	0.001537	SNRPA1, SNRPD3, SNRPD2, HNRNPA1, NAA38, SF3B3, HNRNPU, RBM8A, PCBP1, SNRPB, MAGOHB, SNRNP70, SNRPE	2.898579	0.173673	0.031294	1.760801
**Cardiac muscle contraction**	10	0.001584	ATP1B1, ACTC1, UQCRC1, ATP1A1, UQCRFS1, COX5A, UQCRQ, COX5B, UQCRFSL1, UQCRB, COX6C	3.601785	0.178439	0.027688	1.81371
**Alzheimer’s disease**	15	0.001737	NDUFA5, NDUFA2, UQCRC1, ATP5F1, COX5A, UQCRFS1, COX5B, UQCRQ, UQCRFSL1, COX6C, NDUFS7, NDUFS6, NDUFS4, NDUFV1, NDUFS1, UQCRB	2.58533	0.193896	0.026583	1.987221

**Table 2:** Proteins identified in PI3K/Akt/mTOR, p38 MAPK, Apoptosis, and wnt signaling pathways The protein name, gene symbol, accession number, and PubMed identifier (PMID) for the journal linking the protein to a particular signaling pathway are listed for each protein.

Protein Name	Gene symbol	PMID

***MAPK (pERK) Pathway***		
Fascin	FSCN1	20502940
Galectin-7	LGALS7	21289092
Lumican	LUM	23154825
Serpin H1	SERPINH1	20188343
Guanine nucleotide-binding protein subunit beta-2-like 1	GNB2L1	22240482
Collagen alpha-2(I) chain	COL1A2	22131293
UDP-glucose 6-dehydrogenase	UGDH	14505572
Metalloproteinase inhibitor 1	TIMP1	23555182
Carboxypeptidase E OS	CPE	22824791
Hepatoma-derived growth factor-related protein 3	HDGFRP3	22490522
Ras-related protein Rab-	RAB7A	19372461
ATP synthase subunit e, mitochondrial OS	ATP5I	11939412
Chromogranin-A OS	CHGA	10197763
Galectin-3-binding protein	LGALS3BP	22389450
Peroxiredoxin-6	PRDX6	21346153
Stress-70 protein, mitochondrial	HSPA9	12646231
Annexin A2 OS	ANXA2	22040021
Peroxiredoxin-1	PRDX1	23602274
RasGAP-like with IQ motifs	IQGAP1	23603816
Tripeptidyl-peptidase 1	TPP1	22101936
Filamin-A	FLNA	22203038

***Apoptosis***		
FACT complex subunit SSRP1	SSRP1	16498457
Apoptosis-inducing factor 1, mitochondrial OS	AIFM1	16636662
Cytochrome c oxidase subunit 6C	COX6C	22860893
Diablo homolog, mitochondrial	DIABLO	10972280
Eukaryotic translation initiation factor 3 subunit B	EIF3B	22234522
Glutaredoxin-1	GLRX	17185628
Glutathione peroxidase 3	GPX3	22461624
Heterogeneous nuclear ribonucleoprotein U	HNRNPU	20101230
Mitochondrial carrier homolog 2	MTCH2	15899861
Profilin-1	PFN1	23331014
Rho GDP-dissociation inhibitor 1	ARHGDIA	19077262
Sorcin	SRI	22052463
Very long chain acyl-CoA dehydrogenase, mitochondrial	ACADVL	9680378
Polyadenylate-binding protein 2	PABPN1	22519734
Galectin-7	LGALS7	21289092
DNA replication licensing factor MCM3	MCM3	10495426
Annexin A2 OS	ANXA2	22040021

***PI3K/Akt/mTOR Pathway***		
Basal cell adhesion molecule OS	BCAM	23160466
Peroxiredoxin-1	PRDX1	19941984
N(G), N(G)-dimethylarginine dimethylaminohydrolase1	DDAH1	21212404
Proliferating cell nuclear antigen	PCNA	23298485
Filamin-A	FLNA	22203038
Aldo-keto reductase family 1 member C3 OS	AKR1C3	18508192
ATP-citrate synthase OS	ACLY	18922930
Stathmin	STMN1	21683992
Serum amyloid P-component	APCS	23182717
UDP-glucose 6-dehydrogenase	UGDH	14505572
Keratin, type II cytoskeletal 8	KRT8	23449973
Metalloproteinase inhibitor 1	TIMP1	23555182
Collagen alpha-1(VI) chain	COL6A1	11279127
Collagen alpha-3(VI) chain	COL6A3	11279127
Heterogeneous nuclear ribonucleoprotein F	HNRNPF	21157483
L-lactate dehydrogenase B chain	LDHB	21199794
Poly [ADP-ribose] polymerase 1	PARP1	17525332
Small nuclear ribonucleoprotein E	SNRPE	23358685
Guanine nucleotide-binding protein subunit beta-2-like 1	GNB2L1	22240482

***Wnt Pathway***		
Carboxypeptidase E OS	CPE	22824791
ATP-dependent RNA helicase DDX3X	DDX3X	23413191
High mobility group protein B2*	HMGB2	19805379
Guanine nucleotide-binding protein subunit beta-2-like 1	GNB2L1	22240482
Glutathione S-transferase Mu 3	GSTM3	20118494
Methyl-CpG-binding protein 2	MECP2	23200852
Moesin	MSN	23221384
Ras GTPase-activating protein-binding protein 1	G3BP1	21652632
Annexin A1 OS	ANXA1	21383699
